# Frequency division denoising algorithm based on VIF adaptive 2D-VMD ultrasound image

**DOI:** 10.1371/journal.pone.0248146

**Published:** 2021-03-10

**Authors:** Hongbo Yan, Pengbo Zhao, Zhuang Du, Yang Xu, Pei Liu

**Affiliations:** 1 School of Mechanical Engineering, Inner Mongolia University of Science and Technology, Baotou, Inner Mongolia, China; 2 The First Affiliated Hospital of Baotou Medical College, Inner Mongolia University of Science and Technology, Baotou, Inner Mongolia, China; Vellore Institute of Technology, INDIA

## Abstract

Ultrasound imaging has developed into an indispensable imaging technology in medical diagnosis and treatment applications due to its unique advantages, such as safety, affordability, and convenience. With the development of data information acquisition technology, ultrasound imaging is increasingly susceptible to speckle noise, which leads to defects, such as low resolution, poor contrast, spots, and shadows, which affect the accuracy of physician analysis and diagnosis. To solve this problem, we proposed a frequency division denoising algorithm combining transform domain and spatial domain. First, the ultrasound image was decomposed into a series of sub-modal images using 2D variational mode decomposition (2D-VMD), and adaptively determined 2D-VMD parameter K value based on visual information fidelity (VIF) criterion. Then, an anisotropic diffusion filter was used to denoise low-frequency sub-modal images, and a 3D block matching algorithm (BM3D) was used to reduce noise for high-frequency images with high noise. Finally, each sub-modal image was reconstructed after processing to obtain the denoised ultrasound image. In the comparative experiments of synthetic, simulation, and real images, the performance of this method was quantitatively evaluated. Various results show that the ability of this algorithm in denoising and maintaining structural details is significantly better than that of other algorithms.

## Introduction

In the medical field, the rapidly developing ultrasound technology has become one of the most important imaging forms. Compared with CT, X-ray, MRI, PET and other imaging technologies, advantages of ultrasound technology include its high safety, absence of radioactivity, and low cost. It has been widely used in clinical diagnosis and treatment [1,2]. It plays an irreplaceable role in breast, abdominal organs, and fetal development of pregnant women [3–5].

With the development of engineering technology, people apply the dynamic frequency scanning technology to ultrasound imaging instruments to improve the image quality of ultrasound imaging. It can automatically switch the working frequency according to the depth of the detection target, which improves the penetration depth of the detection while ensuring the resolution of ultrasound imaging [6]. However, in the acquisition and transmission of the ultrasound image, the acoustic impedance of various human tissues is uneven and the spatial distribution is random, causing the scattering particles to interfere with one another; this phenomenon, coupled with the frequent switching of the working frequency, causes speckle noise with different brightness to form easily in the image [7]. Speckle noise considerably reduces the image quality, blurs the edge details, seriously affects the identification and positioning of the lesion area, and complicates the inspection of the subtle lesions; as such, the accuracy of image feature extraction, segmentation, registration, and classification is reduced [8,9], thus increasing the difficulty of medical diagnosis and treatment. Therefore, finding an effective method of denoising medical ultrasound images [10,11] has become a challenge in current research. Many medical ultrasound image denoising methods have been continuously proposed. Two types of denoising methods are commonly used. One type based on the spatial domain includes median filter [12], Lee filter [13], Kuan filter [14], Frost filter [15], OBNLM [16], SRAD [17], and BM3D [18]. This kind of processing algorithm directly filters the image, these algorithms are directly applied to the original image for denoising processing, in order to obtain better noise removal information, the edges of the image are weakened, many high-frequency information such as the fine structure of the image is filtered out, and the resolution of the image is reduced. The other type is based on the transform domain, which uses mathematical transformation to convert the image from the transformation domain to the frequency domain before denoising; examples include wavelet transform and Fourier transform [19,20]. Wavelet transform has a good denoising effect, but the algorithm itself can easily cause blurred edges and involve the setting up of many parameters.

In 2014, Dragomiretskiy et al. proposed Variational Mode Decomposition (VMD) [21], a new adaptive signal decomposition method. It addresses the shortcomings of traditional signal decomposition methods and is widely used in mechanical fault diagnosis and feature extraction [22,23]; they extended the VMD algorithm to the 2D field. In recent years, 2D-VMD [24] was introduced into image processing and has become a novel research direction. Suseelan et al. [25] proposed a method to effectively defog a single image based on 2D-VMD. It can recognize and remove sub-modal images containing haze, achieve a dehazing effect, and completely retain the edge information; U. Raghavendra et al. [26] applied 2D-VMD to the texture feature extraction of ultrasound images, analyzed the structural changes between the images after decomposition, and filtered out the ultrasound images of heart failure; The above-mentioned scholars used this decomposition method well to retain the image structure information, but it has not been applied to image denoising. Wei Z [27] successfully applied 2D-VMD to the road surface image denoising field of road engineering, retaining the original image information and improving the signal-to-noise ratio. Xiao et al. [28] proposed a method of combining 2D variational modal decomposition and mutual information to denoise the Digital Speckle Pattern Interferometry (DSPI) phase map, adaptively extracting noise-free components to achieve the denoising effect. Liu et al [29] proposed a denoising method of 2D-VMD and adaptive median filtering. After the image was decomposed, only mode one was selected as the image for subsequent denoising processing, which achieved improved results, but discarded some high-frequency sub-modes of edge information. Most scholars in the above-mentioned literature fully used the advantages of 2D-VMD adaptive decomposition, which can effectively extract the feature information in the decomposed sub-image, and can separate the noise to the greatest extent for processing, and retain the original image information. However, when processing sub-modal images, some researchers disregard or discard images that contain high-frequency information, resulting in the loss of detailed information. In the decomposition process, 2D-VMD, similar to VMD, also has problems of over decomposition and under decomposition (the value of K) [30]. However, none of the above-mentioned scholars gave a detailed explanation on the selection of the parameter K value and only used empirical methods to determine or directly use the default value.

At present, the application of 2D-VMD on ultrasonic image denoising processing is not well studied. In response to the above problems, this paper proposes an algorithm combining frequency division in transform domain and denoising in spatial domain. First, the ultrasound image was decomposed into sub-modal images of different frequency bands using 2D-VMD, and the VIF difference between the sub-modal images is calculated. For the high-frequency sub-modal images with considerable noise, BM3D is used for denoising, and the denoised sub-modal images are reconstructed. Based on the difference in the iterative operation, the appropriate K value is selected adaptively, and in the spatial domain, the low-frequency sub-modal image is subjected to anisotropic diffusion filtering for denoising, and the final image is obtained. Finally, compare and verify the synthetic image, simulated image and real image. The experimental results show that this algorithm is better than other algorithms, while fully denoising, it preserves the details of the image as much as possible.

The rest of this article is organized as follows: the second section introduces materials and methods, including noise model, 2D-VMD, anisotropic diffusion filter, and BM3D; the third section describes in detail the use of VIF to adaptively determine the parameter K value in 2D-VMD; and the fourth section simulation and experimental research and discussion. The final summary is in Section 5.

## Materials and methods

### Speckle noise model

At present, the speckle noise in ultrasound images can be described as a multiplicative noise model [7,31,32].

g(x)≈f(x)η(x) (1)

where *x* is the pixel position, *g*(*x*) is the observed image, *f*(*x*) is the original image, and *η*(*x*) is a Gaussian noise.

Through logarithmic transformation, the above equation is converted into additive noise that is convenient to handle, and the following equation is obtained:

log(g(x))≈log(f(x))+log(η(x)) (2)

Many studies have shown that the standard speckle noise model can be accurately defined as speckle noise in ultrasound images [33], and it has been widely used by many scholars [31–33]. This model is shown in the following formula:

$$g\left(x\right)=f\left(x\right)+f{\left(x\right)}^{n}\eta \left(x\right)$$ (3)

Where factor is a parameter that defines the type of medical ultrasound image and is a zero mean Gaussian distribution. In ultrasound image study, *n* is set to 0.5 because it represents ultrasound data well. It is most suitable for ultrasound noise images. When *n* is equal to 1, the model is the multiplicative noise. Therefore, in subsequent experiments, the above model is considered to add noise.

## 2D-VMD theory

In medical ultrasound examination and diagnosis, the dynamic frequency scanning technology relies on multi-frequency simultaneous transmission and reception of probes and variable passband filters to realize the detection of superficial tissues and automatically adopt high operating frequencies. This technology is in contrast with traditional ultrasound instruments that require manual probe replacement, and can work with the increase of tissue depth, resulting in the frequency to drop automatically. The multi-frequency simultaneous transceiver probe can transmit sound waves with different frequency ranges at the same time. The frequency range can reach 1.8–12 MHz, and the variable passband filter can automatically adjust the passband according to depth. The echo signal in a narrow frequency range should be selectively accepted and amplified, and the image fusion technology should be applied to fuse the far-field image of the low-frequency signal and the near-field image of the high-frequency signal into an ultrasound image, which can easily cause noise overlap and accumulate.

2D-VMD was used to decompose and process the image. The overall framework of decomposition is to construct and solve the mutated problem. We updated the bandwidth of each mode to minimize the sum of all bandwidths and obtained the center frequency of each mode and the corresponding mode [24]. 2D-VMD was used to decompose the ultrasound image non-recursively and adaptively into sub-modal images of different frequency bands, treat the retained detailed image information as low-frequency sub-images, and treat the images containing significant noise and edge information as high-frequency images for sub-modal images. The two types of images were separated and denoised separately.

In the 2D case, a half-plane of the frequency domain was set to zero, which is equivalent to a vector, denoted as *ω*_*k*_. The 2D analytical signal is defined in the frequency domain as [24].

$${\hat{u}}_{AS,k}(\vec{\omega })=\{\begin{array}{ll} 2{\hat{u}}_{k}(\vec{\omega }), & \mathrm{if}\;\vec{\omega }\cdot {\vec{\omega }}_{k}>0\\ {\hat{u}}_{k}(\vec{\omega }), & \mathrm{if}\;\vec{\omega }\cdot {\vec{\omega }}_{k}=0\\ 0, & \mathrm{if}\;\vec{\omega }\cdot {\vec{\omega }}_{k}<0 \end{array}$$ (4)

According to the frequency domain definition and Fourier transform characteristics of the 2D analytical signal, the relationship between the 2D analytical signal *u*_*AS*,*k*_(*x*) and the modal function *u*_*k*_(*x*) was obtained as follows:

$${\hat{u}}_{AS,k}\left(\vec{\omega }\right)=u_{k}\left(\vec{x}\right)*\left[\delta \left(\langle \vec{x},{\vec{\omega }}_{k}\rangle \right)+\frac{i}{\pi \left(\vec{x},{\vec{\omega }}_{k}\right)}\right]*\left(\langle \vec{x},{\vec{\omega }}_{k,⊥}\rangle \right)$$ (5)

Where * indicates convolution and *ω*_*k*_ indicates the reference direction for calculating the analytical signal. The corresponding constrained mutated model can be expressed as

$$\min\left\{{\sum_{k}\alpha_{k}\left\|\nabla \left[u_{AS,k}\left(\vec{x}\right)e^{-j\langle {\vec{\omega }}_{k},{\vec{x}}_{k}\rangle }\right]\right\|}_{2}^{2}\right\}$$

$$s.t.\;\sum_{k}u_{k}=f$$ (6)

The constrained mutated of bandwidth was converted into an unconstrained mutated solution:

$$L\left(\left\{u_{k}\right\},\left\{\omega_{k}\right\},\lambda \right)≔{\sum_{k}\alpha_{k}\left\|\nabla \left[u_{AS,k}\left(x\right)e^{-j\langle \omega_{k},t\rangle }\right]\right\|}_{2}^{2}+{\left\|f\left(x\right)-\sum_{k}u_{k}\left(x\right)\right\|}_{2}^{2}+\langle \lambda \left(x\right),f\left(x\right)-\sum_{k}u_{k}\left(x\right)\rangle$$ (7)

Where *α* is the penalty factor and *λ* is the Lagrangian multiplier. The alternating direction method of multiplication operator was used to solve the above mutated problem and obtain the saddle point of the above formula by alternately updating $u_{k}^{n+1}$, $\omega_{k}^{n+1}$, $\lambda_{k}^{n+1}$. Pasval Fourier isometric transform was used for conversion to the frequency domain

$$\begin{array}{l} {\hat{u}}_{k}^{n+1}\left(\vec{\omega }\right)=\left(\hat{f}\left(\vec{\omega }\right)-\sum_{i\neq k}{\hat{u}}_{k}\left(\vec{\omega }\right)+\frac{\hat{\lambda }\left(\vec{\omega }\right)}{2}\right)\frac{1}{1+2\alpha {\left|\vec{\omega }-{\vec{\omega }}_{k}\right|}^{2}}\\ \;\forall \vec{\omega }\in \Omega_{k}:\Omega_{k}=\left\{\vec{\omega }|\vec{\omega }\cdot {\vec{\omega }}_{k}\geq 0\right\} \end{array}$$ (8)

Similarly, the update formula of ${\vec{\omega }}_{k}^{n+1}$ frequency domain can be obtained as follows:

$${\vec{\omega }}_{k}^{n+1}=\frac{{\int_{\Omega_{k}}\vec{\omega }\left|{\hat{u}}_{k}\left(\vec{\omega }\right)\right|}^{2}d\vec{\omega }}{{\int_{\Omega_{k}}\left|{\hat{u}}_{k}\left(\vec{\omega }\right)\right|}^{2}d\vec{\omega }}$$ (9)

Where ${\hat{u}}_{k}^{n+1}\left(\omega \right)$ is the result of Wiener filtering, $\omega_{k}^{n+1}$ is the center of gravity of the power spectrum of the modal function; After performing the inverse Fourier transform on $\left\{{\hat{\omega }}_{k}\left(\omega \right)\right\}$, the corresponding real part {*u*_*k*_(*t*)} is obtained.

The specific process steps of the 2D-VMD algorithm are as follows [24]:

Initialize n = 0 for $\left\{{\hat{u}}_{k}^{0}\right\}$、 $\left\{{\hat{\omega }}_{k}^{0}\right\}$、 $\left\{{\hat{\lambda }}_{k}^{0}\right\}$.

Update according to the following formula *u*_*k*_ in the frequency domain:

$$\begin{array}{l} {\hat{u}}_{k}^{n+1}\left(\vec{\omega }\right)=\left(\hat{f}\left(\vec{\omega }\right)-\sum_{i\neq k}{\hat{u}}_{k}\left(\vec{\omega }\right)+\frac{\hat{\lambda }\left(\vec{\omega }\right)}{2}\right)\frac{1}{1+2\alpha {\left|\vec{\omega }-{\vec{\omega }}_{k}\right|}^{2}}\\ \;\forall \vec{\omega }\in \Omega_{k}:\Omega_{k}=\left\{\vec{\omega }|\vec{\omega }\cdot {\vec{\omega }}_{k}\geq 0\right\} \end{array}$$

Update *ω*_*k*_ according to ${\vec{\omega }}_{k}^{n+1}=\frac{{\int_{\Omega_{k}}\vec{\omega }\left|{\hat{u}}_{k}\left(\vec{\omega }\right)\right|}^{2}d\vec{\omega }}{{\int_{\Omega_{k}}\left|{\hat{u}}_{k}\left(\vec{\omega }\right)\right|}^{2}d\vec{\omega }}$

Update value *λ*, where ${\hat{\lambda }}^{n+1}\left(\omega \right)\leftarrow {\hat{\lambda }}^{n}\left(\omega \right)+\tau \left(\hat{f}\left(\omega \right)-\sum_{k}{\hat{u}}_{k}^{n+1}\left(\omega \right)\right)$

Until $\frac{\sum_{k}{\left\|{\hat{u}}_{k}^{n+1}-{\hat{u}}_{k}^{n}\right\|}_{2}^{2}}{{\left\|{\hat{u}}_{k}^{n}\right\|}_{2}^{2}}<Ke$ is satisfied, the iteration ends.

## Anisotropic diffusion filtering

Traditional filters can remove the noise information in the image effectively and smoothen the image. However, these filters weaken the edge information, and as the number of iterations of the filtering operation continues to increase, the boundary contour may be completely lost. To solve this problem, Perona and Malik proposed anisotropic diffusion filter after exploring the characteristics of the thermal diffusion equation [34]. This filter has good noise resistance and is superior to other methods in preserving the edge information of the original image. It has been widely used in various image processing fields, including medical images [17,35]. Its principle is the treatment of the initial image as a heat field, and each pixel in the image is regarded as a heat flow. According to the gradient value of the current pixel and surrounding pixels and the gradient threshold value to determine whether to diffuse to the surroundings, the neighborhood weighted average is used to removes small gradient changes caused by noise. When a large difference exists between the pixels in a certain area and the current pixels, the pixels in this area may contain the edge information of the picture. The current pixels will not diffuse in this direction, so the edge of the image is preserved while filtering information. After repeated iterations, the noise of the image is removed. For medical ultrasound images, the edge information covers some subtle tissue information, which is extremely important for doctors. The decomposed low-frequency sub-modal image contains most of the image information, and the pixel changes are complex. The filter transforms the corresponding diffusion intensity value according to the size of the pixel gradient value to obtain a better denoising effect. The size refers to the difference between the gradient values of adjacent pixels, according to the difference between this value, change the corresponding diffusion intensity value. The filter selects the corresponding diffusion intensity value according to the difference between the gradient values of adjacent pixels. The edge information is protected, so this method should be considered to denoise the de-noising low-frequency sub-modal image. The denoising model is defined as follows:

$$\begin{array}{l} \partial I\left(x,y\right)=Div\left[c\left(\left|\nabla I\right|\right)\cdot I\right]\\ I\left(x,y,0\right)=I_{0}\left(x,y\right) \end{array}$$ (10)

Where I(x, y, t) is the image signal at time t, div is the divergence operator, ∇ is the

gradient operation, ‖∇‖ is the gradient amplitude, and *c*‖∇‖ is the spread function.

## BM3D theory

BM3D is currently one of the most effective algorithms in image noise reduction. It uses the similarity in the image itself to effectively combine non-local methods and change domain methods. It is applied to ultrasound images, taking advantage of the small change in gray value in the image, Utilizing the characteristics of small gray value changes in ultrasound images and high similarity after image segmentation, applying BM3D to ultrasound images can obtain significant denoising effects and can effectively protect image details [36,37].

**Fig 1:**
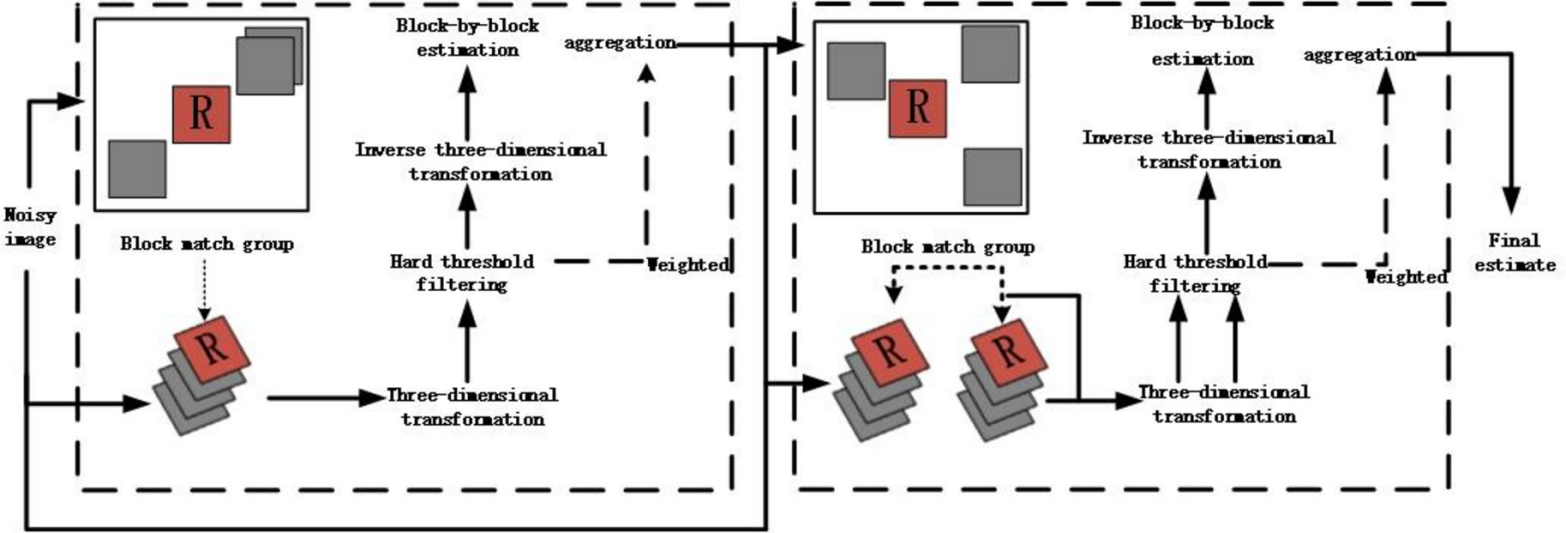
BM3D image denoising flow chart.

The algorithm is divided into two stages: preliminary and final estimation [18]. The first stage obtains a basic estimate of the noisy image: firstly, the image is divided into a number of image blocks of equal size, each image block is found similar image blocks within the set search range, the currently processed block is used as a reference block, after that each reference block and its similar blocks are stacked into a three-dimensional array according to the similarity. The greater the similarity, the closer to the reference block. Then we perform the three-dimensional transform domain hard threshold filter method for the three-dimensional array to reduce the noise, after that we use the three-dimensional inverse transform to obtain the estimated value of the grouped tiles, each similar block may contain multiple estimated values, and the basic estimation of the image is obtained after the weighted average of the multiple estimated values; The second stage is to further improve the denoising performance: firstly the image obtained in the basic estimation are searched for similar blocks to form a new three-dimensional group, Then 3D group is processed by Wiener filtering in 3D transform domain, we perform Wiener filter processing on the 3D group in the basic estimated image corresponding to the original image 3D group, the estimated value of the image is obtained after inverse transformation, and the final estimated value of the image is obtained by weighted average on the overlapping part of the image block. The specific flowchart is as Fig 1.

## Proposed algorithm

**Fig 2:**
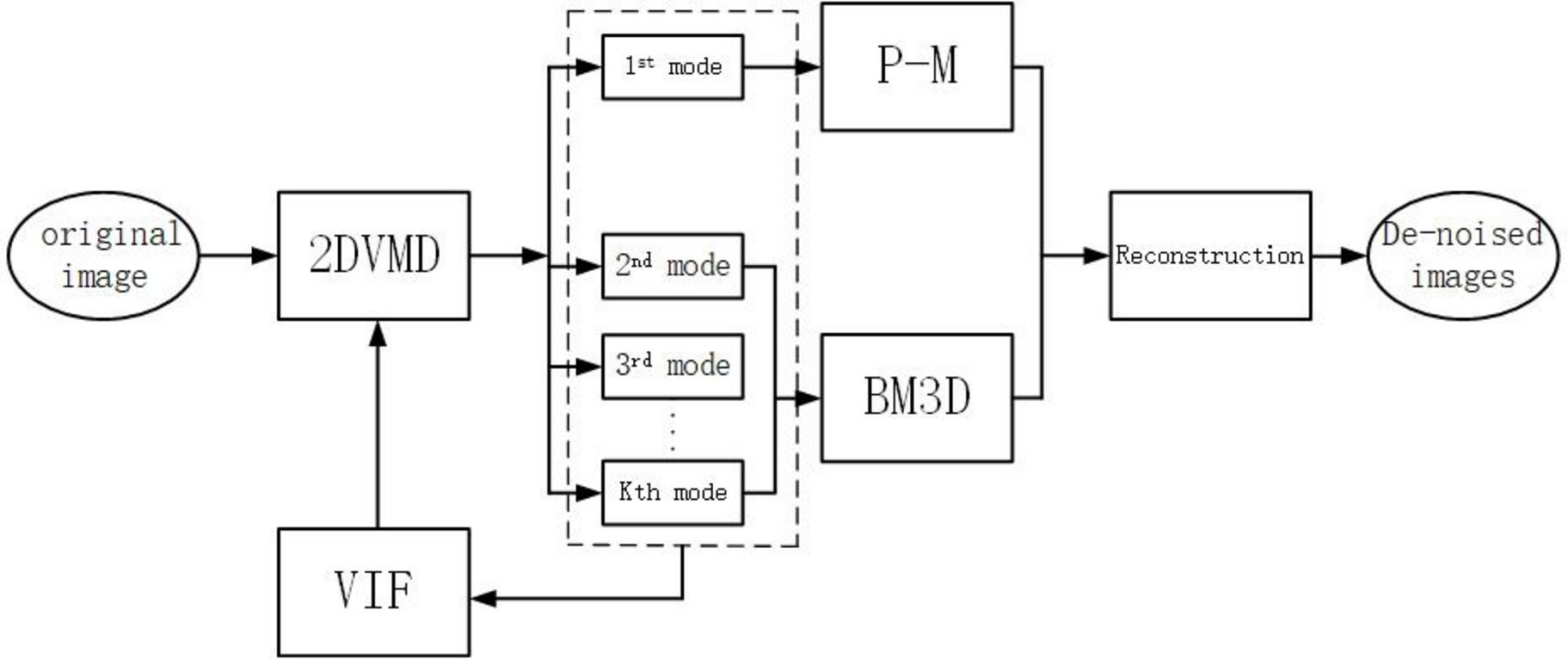
Flow chart of the proposed algorithm.

Based on the above theory, this paper proposes a VIF-based adaptive 2D-VMD frequency division denoising method. The flowchart is as Fig 2.

Step 1: Use 2D-VMD (pre-set a K value) to decompose the acquired medical ultrasound image to obtain K sub-modal images.
Step 2: Calculate the VIF value of each image, and adaptively determine the value of K.
Step 3: In the final decomposed sub-modal image, the first sub-modal image contains more low-frequency information of the image and is processed by denoising with anisotropic diffusion filtering, and the high-frequency image information is decomposed. Use BM3D processing to remove more noise in other sub-images.
Step 4: Finally, reconstruct the processed sub-modal image based on the 2D-VMD reconstruction method, and complete the denoising process to obtain the final denoised image.

## Optimization of the K value

In the research and application of VMD, many scholars have found that the K value is important. When the selected K value is too small, multiple modes of the signal may coexist in the same sub-mode; thus, some information cannot be effectively separated and identified, resulting in under-decomposition [31,38]. Similarly, this kind of problem also exists in 2D-VMD; when K is too small, the decomposition is insufficient, and many high-frequency information cannot be separated reasonably and overlapped in the same sub-modal image, making the subsequent denoising insufficient. When K is too large, it leads to over-decomposition, which causes continuous decomposition at similar center frequencies, and highly similar sub-modal images appear. For the decomposed ultrasound sub-modal images, especially in high-frequency images, the changes in their mutual brightness, distribution, and organizational structure are weak. A method that can calculate the degree of information sharing between the sub-modal images and the original image, judge similar images, select the appropriate K value, and can fully process the K sub-modal images should be sought to prevent blurring of the reconstructed denoising images, and the apperance of ghosting. Therefore, whether the K value is set reasonably or not has a crucial influence on the final denoising result. However, many researchers rarely discuss and study the value of K when using 2D-VMD.

## VIF theory

This work proposes a method for adaptively selecting K values based on VIF and an effective full-reference image quality measurement based on natural scene statistics (NSS) theory, and for estimating the quality of the image by measuring the amount of information shared between the reference image and the test image [39]. First, the natural image is decomposed into several sub-bands, and each sub-band is parsed into blocks (in order to obtain more accurate sharing information between images, this article sets the block size to 3*3). Then, the mutual information in different models of each block is calculated, and each subband is used to measure visual information. Finally, the image quality value is measured by integrating the visual information of all blocks and all sub-bands. Here, VIF combines the following three models [40,41].

The first model is the image source model. The Gaussian scale mixture (GSM) model is an NSS model in the wavelet domain. A GSM is a random field (RF) that can be expressed as a product of two independent RFs and expressed as follows:

$$C=S\cdot U=\left\{S_{i}\cdot U_{i}:i\in I\right\}$$ (11)

Where C denotes the RF of the reference signal in a sub-band, S is an SF of positive scalars, U is a Gaussian vector RF with mean zero and covariance C_U_, and I denotes the set of spatial indices for the RF.

The second is the distortion model. It uses signal attenuation and additive noise model to describe all possible distortion types as follows:

$$D=gC+V=\left\{g_{i}C_{i}+V_{i}:i\in I\right\}$$ (12)

Where D enotes the RF from the corresponding sub-band from the test (distorted) signal; g is a deterministic scalar gain field, and V is a stationary additive zero-mean Gaussian noise RF with variance Cv.

The third is the Human Visual System Model, which models the HVS as a distortion channel limiting the amount of information that could pass through it. The HVS model aims to quantify the uncertainty factors that HVS adds to the image signal. The reference image E and the test image F are modeled by HVS as follows:

E=C+N (13)

F=C+N’ (14)

Among them, E and F denote the visual signal at the output of the HVS model from the reference and the test images in one sub-band, respectively, N and N’ are additive noises and its visual distortion model can be modeled as:

$$C_{N}=C_{N^{\prime }}=\sigma_{n}^{2}I$$ (15)

The mutual information I(C; E) of C and E is calculated to estimate the amount of reference image information that is transmitted to the brain via the HVS channel. The mutual information I(C; F) of C and F is also computed to estimate the amount of distorted image information that is transmitted to the brain through the HVS channel compared with that of the reference image.

$$I\left(C^{N};E^{N}|s^{N}\right)=\frac{1}{2}\sum_{i=1}^{N}\log_{{}_{2}}\left(\frac{\left|s_{i}^{2}C_{U}+\sigma_{n}^{2}I\right|}{\left|\sigma_{n}^{2}I\right|}\right)$$ (16)

Deduced in the same way.

$$I\left(C^{N};F^{N}|s^{N}\right)=\frac{1}{2}\sum_{i=1}^{N}\log_{{}_{2}}\left(\frac{\left|g_{i}^{2}s_{i}^{2}C_{U}+\left(\sigma_{v}^{2}+\sigma_{n}^{2}\right)I\right|}{\left|\left(\sigma_{v}^{2}+\sigma_{n}^{2}\right)I\right|}\right)$$ (17)

Where N represents the number of local blocks in the image band. Based on the above model, the final VIF index is defined as follows:

$$VIF=\frac{\sum_{j=subbands}I\left(C^{N,j};F^{N,j}|s^{N,j}\right)}{\sum_{j=subbands}I\left(C^{N,j};E^{N,j}|s^{N,j}\right)}$$ (18)

## Adaptive selection of K value

**Fig 3:**
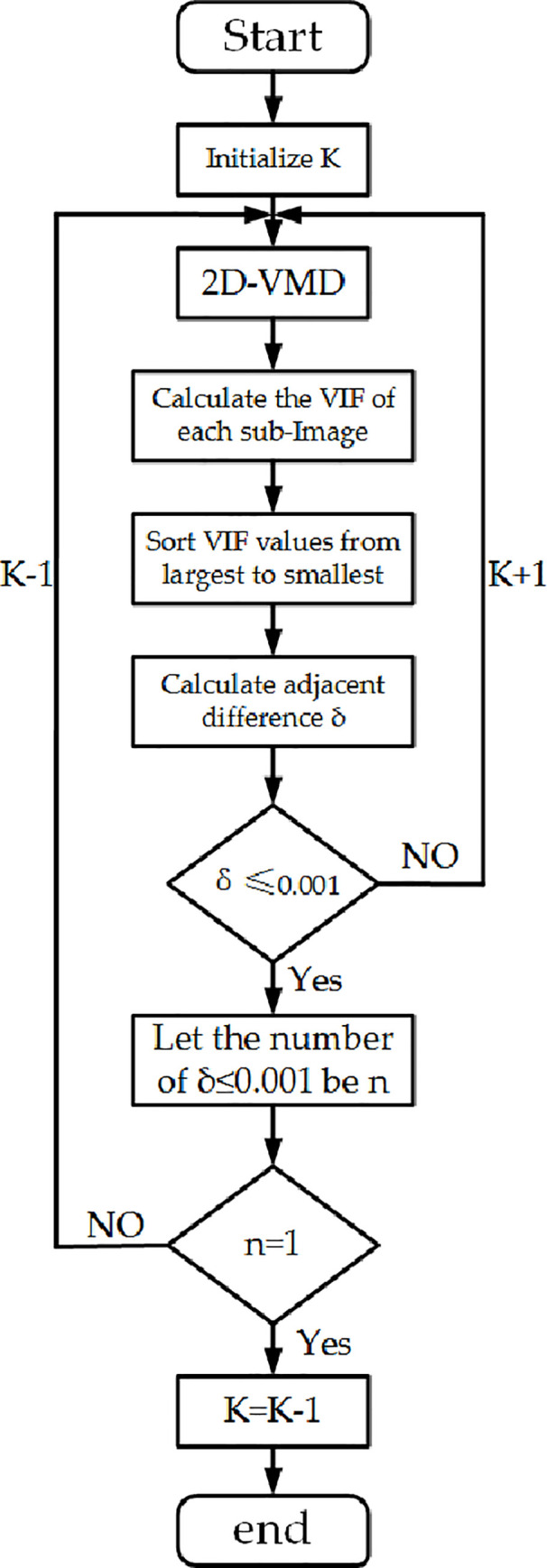
Flow chart of selecting K value.

We propose a method of adaptively determining the K value, based on the characteristics of the decomposed image and the above theory, the flowchart is shown in Fig 3.

Step1: Set an initial K value, use VIF to calculate the decomposed K sub-modal images, and obtain their respective VIF values.
Step2: Sort the series of values and calculate the difference between adjacent values.
Step3: Judge whether *δ* is less than the set threshold of 0.001; if not, increase K by 1 and return to re-decomposition; yes to the next step.
Step4: Calculate the number n of *δ*<0.001, judge whether n is equal to 1; if not, reduce K by 1 and return to the central decomposition; if yes, the output value is K-1, end.

**Fig 4:**
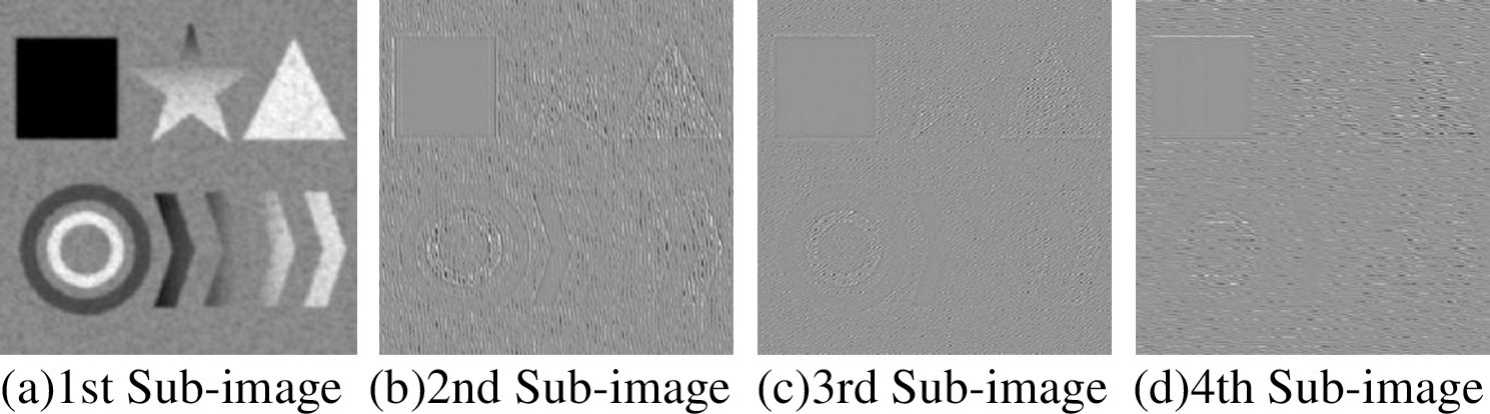
Decomposition effect diagram of composite.

Under different noise levels, many experiments were carried out on different images, and finally, the threshold was determined to be 0.001. The VIF difference between two adjacent images should be less than or equal to 0.001, and the image information between them is similar. For this composite image, the calculated value of K is 4, and the exploded view is shown in Fig 4.

Fig 4 shows that the first sub-modal image is a low-frequency one and contains much image information; other sub-modal images contain much noise and obvious edge structure information, which are high-frequency images. To verify the above K value selection method, within the range of different noise intensity, we decomposed and calculated the synthesized image to obtain the values shown Table 1. Similar values in the table have been marked in bold. When the K value is equal to 5, only one group of adjacent sub-modal images has close VIF values, and the difference is less than or equal to 0.001. When the K value is equal to 6, the adjacent sub-modal images have two groups. The VIF value of the image is close, and the K value of the decomposition of the current composite image is 4, which is consistent with the conclusion drawn by the adaptive calculation.

**Table 1:** VIF value of each sub-modal image.

Noise variance	K	VIF					
0.2	4	0.1389	0.1321	0.0751	0.0715		
	5	0.1518	0.1309	**0.0805**	**0.0799**	0.0740	
	6	0.1509	0.1289	0.0838	**0.0779**	**0.0769**	**0.0761**
0.4	4	0.1138	0.0962	0.0643	0.0620		
	5	0.1229	0.0723	0.0707	**0.0676**	**0.0667**	
	6	0.1087	0.0702	0.0662	**0.0643**	**0.0633**	**0.00628**
0.6	4	0.1146	0.0651	0.0642	0.0594		
	5	0.1089	0.0769	0.0636	**0.0622**	**0.0617**	
	6	0.1085	0.0731	**0.0644**	**0.0641**	**0.0635**	0.0622
0.8	4	0.1076	0.0708	0.0625	0.0617		
	5	0.1021	0.0704	**0.0589**	**0.0585**	0.0573	
	6	0.1009	0.0717	0.0619	**0.0600**	**0.0589**	**0.0589**

### Experimental studies on the proposed method

Starting from the theoretical descriptions in the above sections and according to the characteristics of speckle noise in medical ultrasound images, a denoising algorithm based on adaptive 2D-VMD frequency division combined with spatial technology was proposed; this algorithm is theoretically superior to other algorithms. This section shows the three different types of comparative experiments on synthetic graphs, simulated graphs, and actual graphs conducted further verify the performance of the algorithm.

In the comparison experiment, 10 denoising algorithms (Lee [13], Frost [15], Kuan [14], Wavelet [19], BF [42], Median [12], OBNLM [16], FNLM [43], SRAD [17], and BM3D [18]) were analyzed and compared, and search or experiment was conducted to find the optimal parameters of each comparison algorithm. The window size of the Lee, Frost, Kuan, Median, and BF filters measures 3 × 3.The patch size of the FNLM and OBNLM filters is 5 × 5. The iterations of the SRAD is 200, △*t* = 0.25 for Wavelet, wavelet = ‘coif1’, and Window size 3 × 3; the filter sigma is 20. All algorithms are implemented in MATLAB R2016a on a personal computer with a 2.70 GHz Intel Pentium CPU and 8 GB of RAM.

For quantitative comparison, two objective parameters, the peak signal-to-noise ratio (PSNR) and the structural similarity index measure (SSIM), were used to evaluate and compare various denoising algorithms. PSNR is related to image quality. The larger the PSNR value, the higher the image quality, which means that the image after denoising is closer to the original image as a whole; it can be calculated using the following formula:

$$PSNR=10\times \log_{10}\frac{255^{2}}{\sum_{i=1}^{m}\sum_{j=1}^{n}{\left[f(i,j)-\hat{f}\left(i,j\right)\right]}^{2}}$$ (19)

SSIM is a method that uses the structural information of images to measure the similarity between images [44]. The value range of SSIM is [0,1], which evaluates the relationship between the image and the image in terms of brightness, structure, and contrast. The similarity of the original image, the closer its value is to 1, the more similar the image after denoising is to the original image. The following formula was used for calculation:

$$SSIM\left(f,\hat{f}\right)=\frac{\left(2\mu_{f}\mu_{\hat{f}}+c_{1}\right)\left(2\sigma_{f\hat{f}}+c_{2}\right)}{\left(\mu_{f}^{2}+\mu_{\hat{f}}^{2}+c_{1}\right)\left(\sigma_{f}^{2}+\sigma_{\hat{f}}^{2}+c_{2}\right)}$$ (20)

Among them: *f*(*x*,*y*) represents the original image, and $\hat{f}\left(x,y\right)$ represents the denoised image. *μ*_*f*_ is the average of *f*, ${\hat{\mu }}_{f}$ is the average of $\hat{f}$, $\sigma_{f}^{2}$ is the variance of *f*, $\sigma_{\hat{f}}^{2}$ is the variance of $\hat{f}$, $\sigma_{f\hat{f}}$ is the covariance of *f* and $\hat{f}$. *c*_1_ = (*k*_1_*L*)^2^ and *c*_2_ = (*k*_2_*L*)^2^ are used to maintain the stability constant, and *L* is the dynamic range of the pixel, *k*_1_ = 0.01, *k*_2_ = 0.03.

## Synthetic image experiment

**Fig 5:**
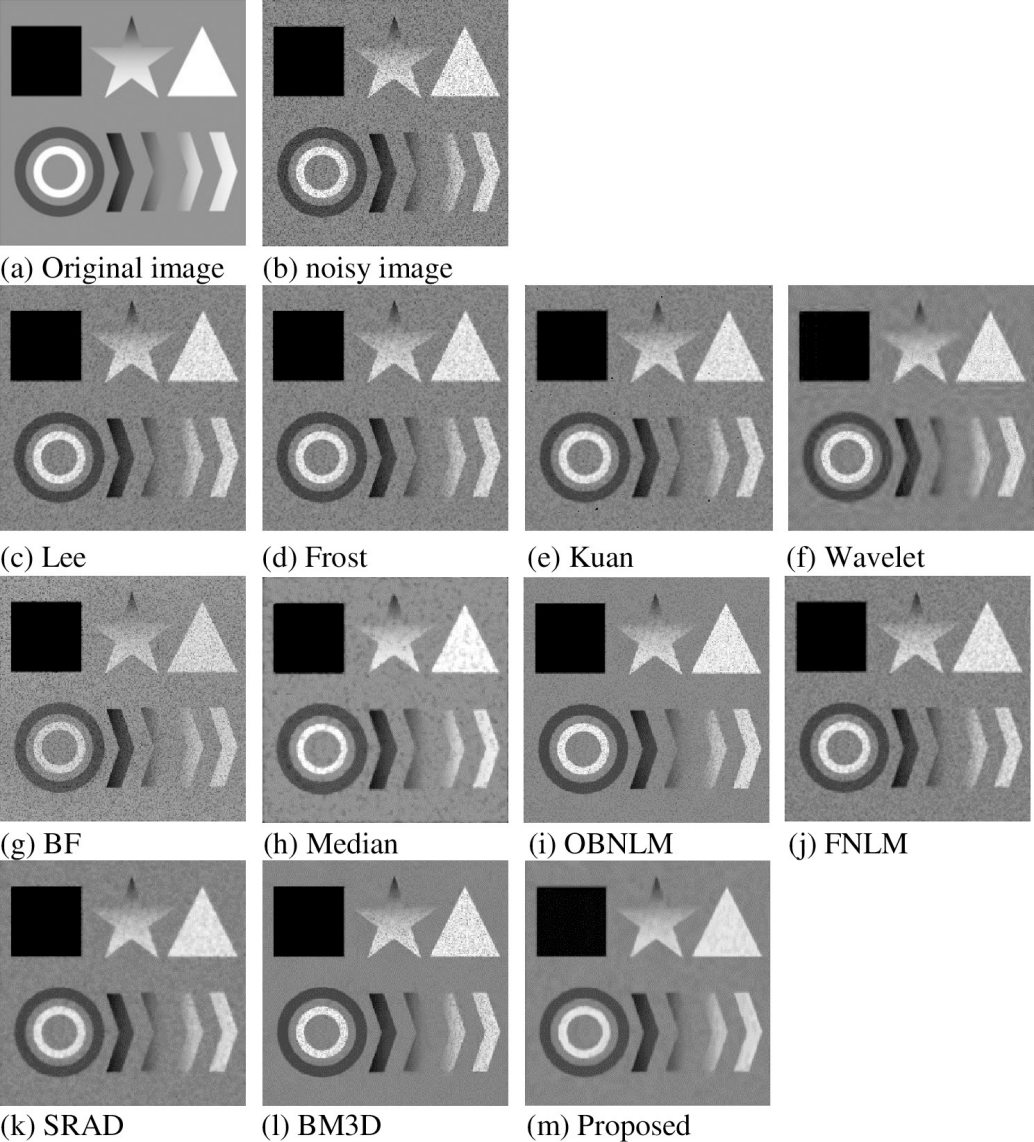
Composite image denoising effect diagram (variance 0.6).

To fully verify the performance of the proposed denoising algorithm objectively, we considered including circular arc, angle, pixel value, and edge changes in the composite image, and we increased the complexity of image information as much as possible to ensure the validity of the experiment and comprehensiveness. The size of the composite image is 302*302. For this noise-free composite image, the added noise comes from the multiplicative noise model mentioned in section 2.1. The different algorithms mentioned above were used to denoise separately, and the results obtained were compared and analyzed. Fig 5 only shows a comparison of denoising effects with a variance of 0.6 given the space limitation.

As shown in Fig 5, under the noise variance of 0.6, Fig 5(C), 5(D), 5(E) and 5(G) are not effective in denoising, and the denoised image still contains much noise. The protection of the original image structure information is not good; the wavelet coefficients in the wavelet high-frequency sub-band are mistaken for the noise coefficient and removed. A small amount of noise remains in Fig 5(F), and the details, such as edges and textures, are lost. SRAD contains a small amount of noise but blurs much image information, and the corners and edge points have been rounded. The median filter retains part of the edge information. In the denoising process, the image information and noise are blurred and shaped like snowflakes, and the overall image is not clear. The OBNLM and FNLM methods have insufficient denoising ability under strong noise. Much noise is shown in Fig 5(I) and 5(J). Compared with OBNLM, FNLM destroys the structural features of the original image. BM3D has strong denoising ability. Fig 5(L) has a good visual effect but shows obvious noise on the graph. Noise is not obvious in Fig 5(M). The details are more complete and clearer, and the more texture information is shown compared with the contrast method.

**Fig 6:**
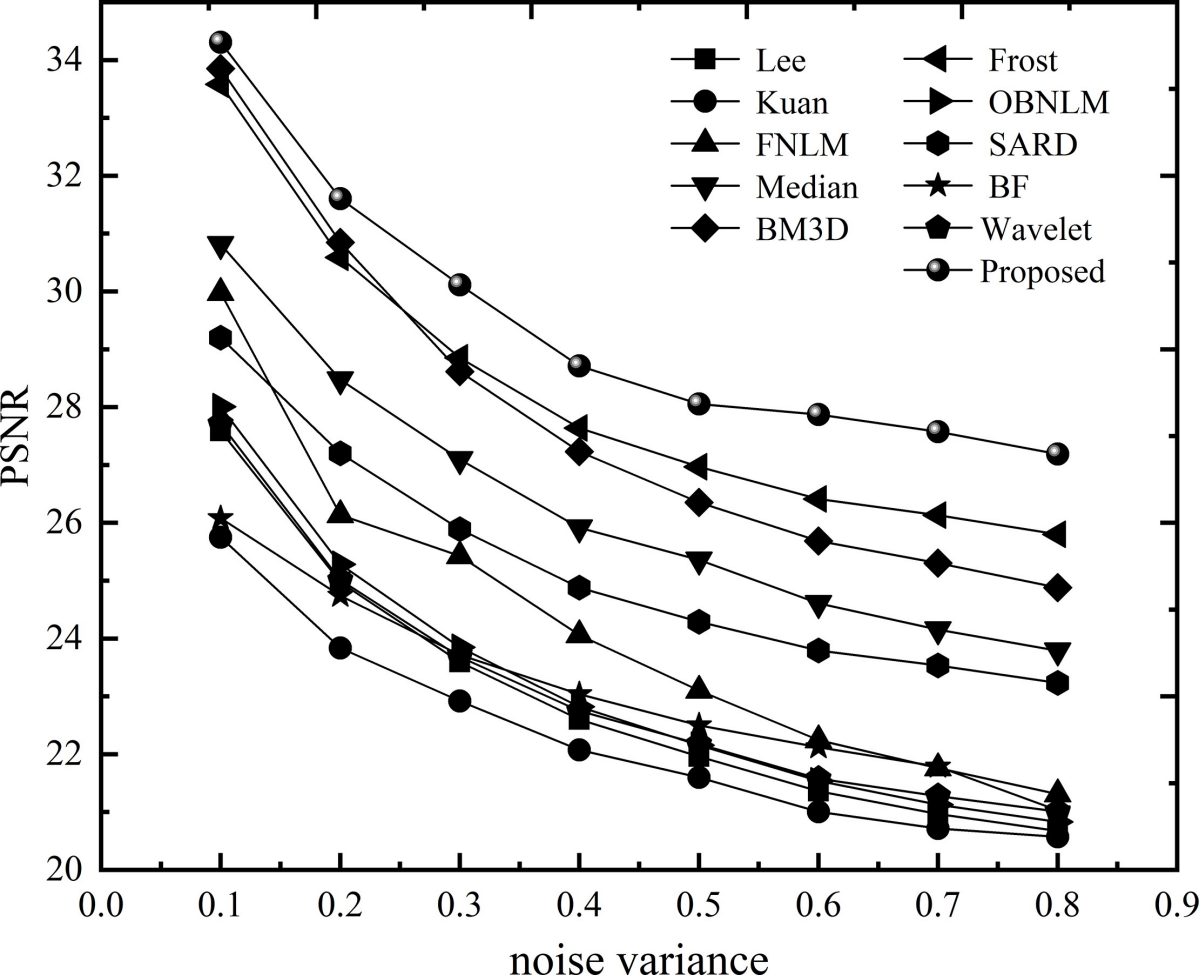
PSNR value of each algorithm under different noise.

**Fig 7:**
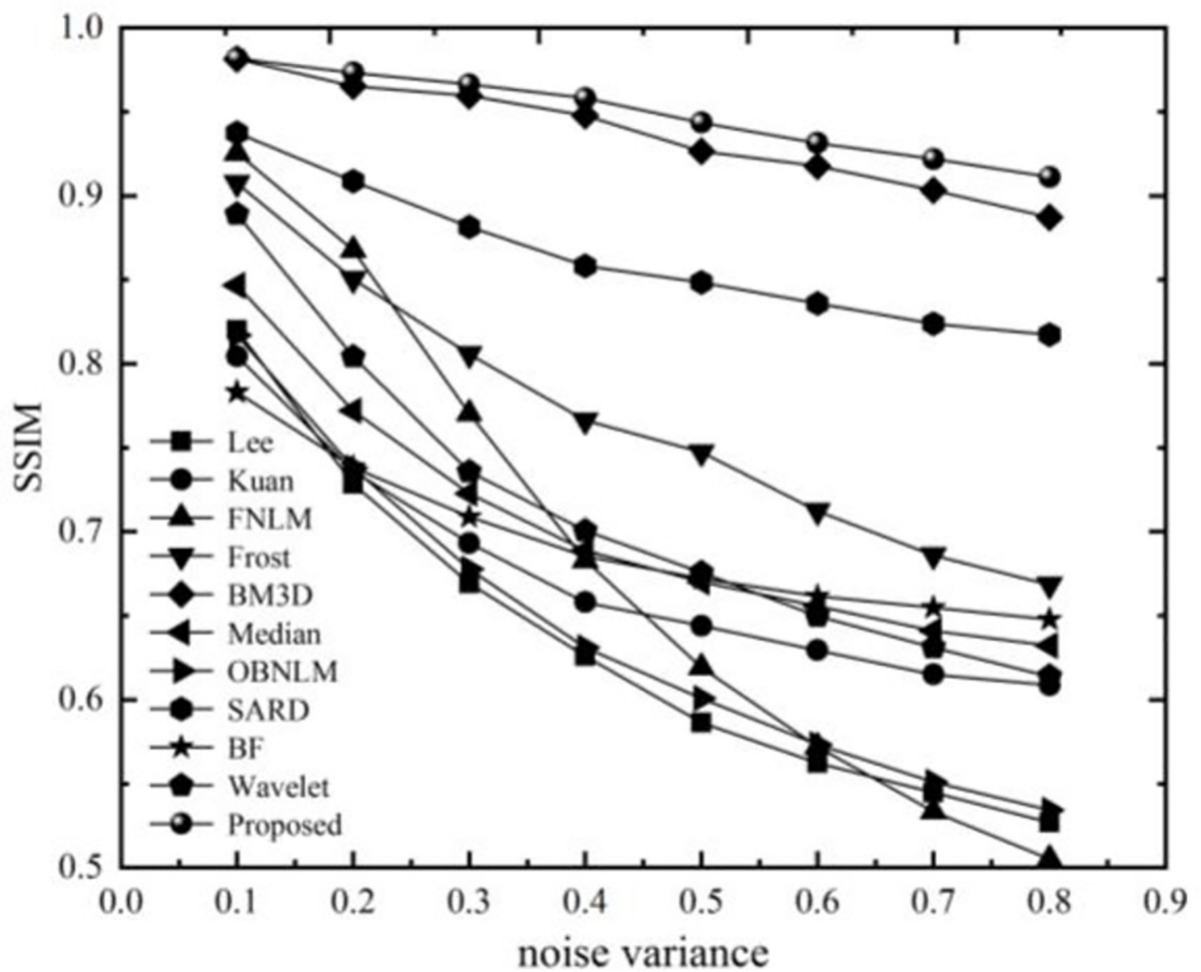
SSIM value of each algorithm under different noises.

In order to further test the performance of the method proposed in this article, we considered conducting comparative experiments under different noise variance conditions to further test the performance of the method proposed in this article. The noise variance range is 0.1–0.8 with an interval of 0.1. The experimental results are shown in Figs 6 and 7.

Fig 6 shows that in different noise environments, the method proposed in this paper has the highest values among all methods. In the low noise range, the variance is less than 0.2, and BM3D and FNLM are not much different from the proposed method. With increasing noise, the denoising performance of the method in this paper gradually becomes prominent, which is far better than that of the other methods. Under the strong noise level, the PSNR value of this method can be maintained above 27dB, which has a good visual effect, and the maximum difference between other methods is approximately 7dB. Fig 7 shows that the SSIM value of the method proposed in this article is always higher than that of other methods, showing that the denoised image has a similar structure and performs well in terms of structural feature retention. Although the BM3D and the current method have close values when the noise variance is less than 0.4, the advantages of the current method becomes apparent after the noise continues to increase, and the gap between the two gradually increases. As far as other methods are concerned, the structure retention performance is still better at low noise, but as the noise becomes stronger, the ability to maintain characteristics is reduced while blindly pursuing the denoising effect, which cannot achieve the best balance.

## Ultrasound simulation image experiment

To further simulate ultrasound images, we used a simulated kidney image created from the Jensen Field II program by using Tupholme–Stepanishen method [45]. This phantom can be downloaded from Jensen’s website (http://field-ii.dk/). The following fig shows the denoising effect of different methods, and Table 2 shows the comparison of the PSNR and SSIM indicators of each method.

**Table 2:** Denoising PSNR and SSIM values of each method are relative to the simulation diagram.

Methods	PSNR	SSIM
Lee	15.4166	0.3896
Frost	15.3696	0.36
Kuan	15.3064	0.3009
OBNLM	14.6619	0.3012
FNLM	15.3055	0.306
SRAD	15.4716	0.3906
BF	13.9911	0.194
Wavelet	14.8351	0.1722
Median	15.3842	0.3946
BM3D	15.5129	0.4132
Proposed	**16.1197**	**0.4464**

**Fig 8:**
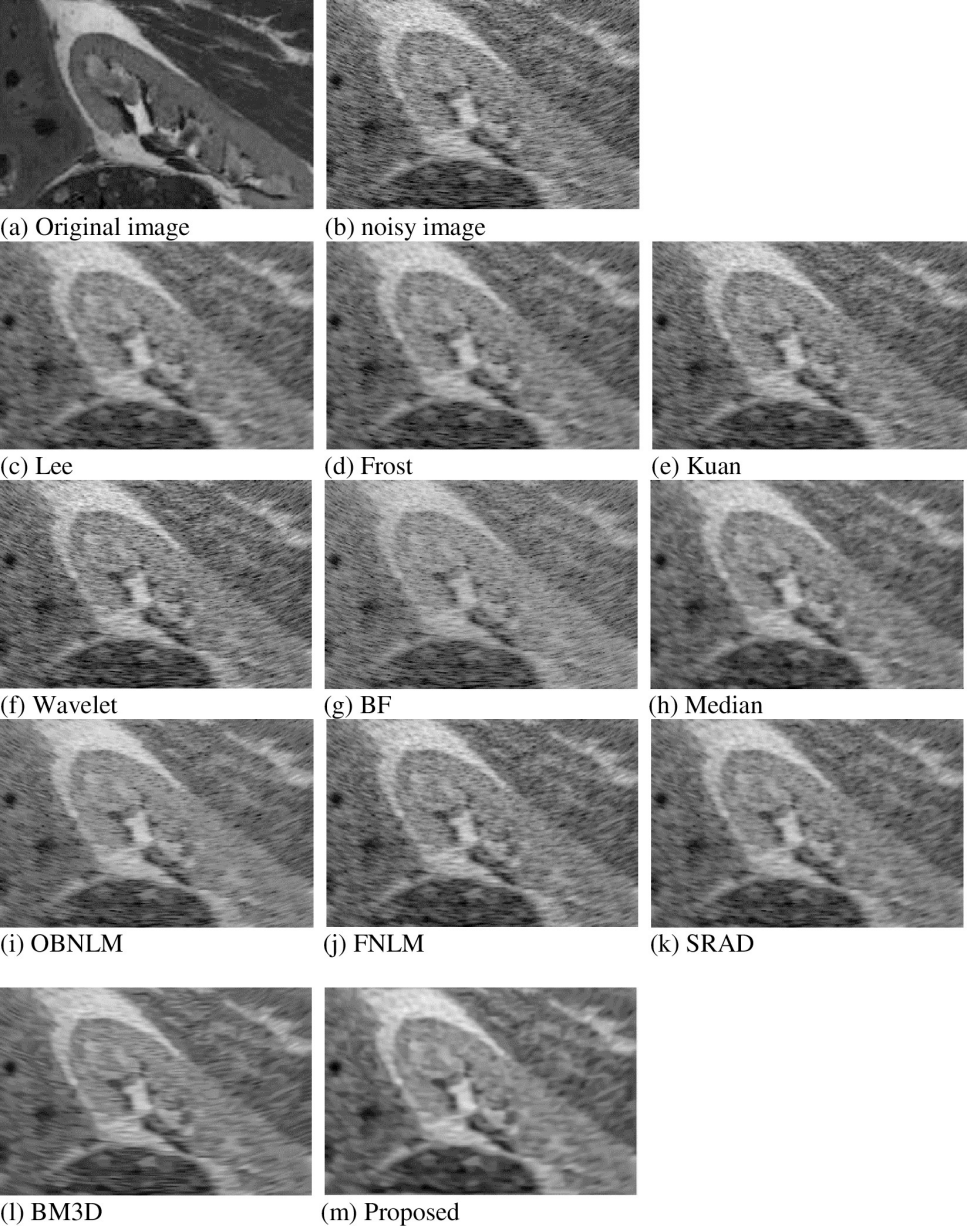
Denoising comparison of simulation diagram.

From the third section, the parameter value K is 3 when the simulation image is decomposed. From the visual effect, Fig 8(A) is the original ultrasound kidney image, and Fig 8(B) is the image obtained after adding noise. Fig 8(C)–8(M) show the denoising effect of different methods. Fig 8(D) and 8(F) have remarkable noise, and the removal effect is not good. Fig 8(I) and 8(M) are compared with other images, and their noise reduction is obvious, and part of the outline appears. The difference in denoising effects of other images is almost invisible to the naked eye.

The performance parameters in Table 2 show that the PSNR value of the method proposed in this article is more than 16, ranking first. The PSNR value of BF, Wavelet, and OBNLM is less than 15, and the denoising ability is not good. The values of other methods are between 15–16, with a slight difference.

## Real image experiment

This work aims to evaluate the performance of the filter in real scenes. Therefore, we considered different methods to denoise clinical medical ultrasound images, including the fetus, gall bladder, breast, and kidney. All clinical images used were provided by the First Affiliated Hospital of Baotou Medical College, Inner Mongolia University of Science and Technology, and the patients participating in our study provided written consent.

**Fig 9:**
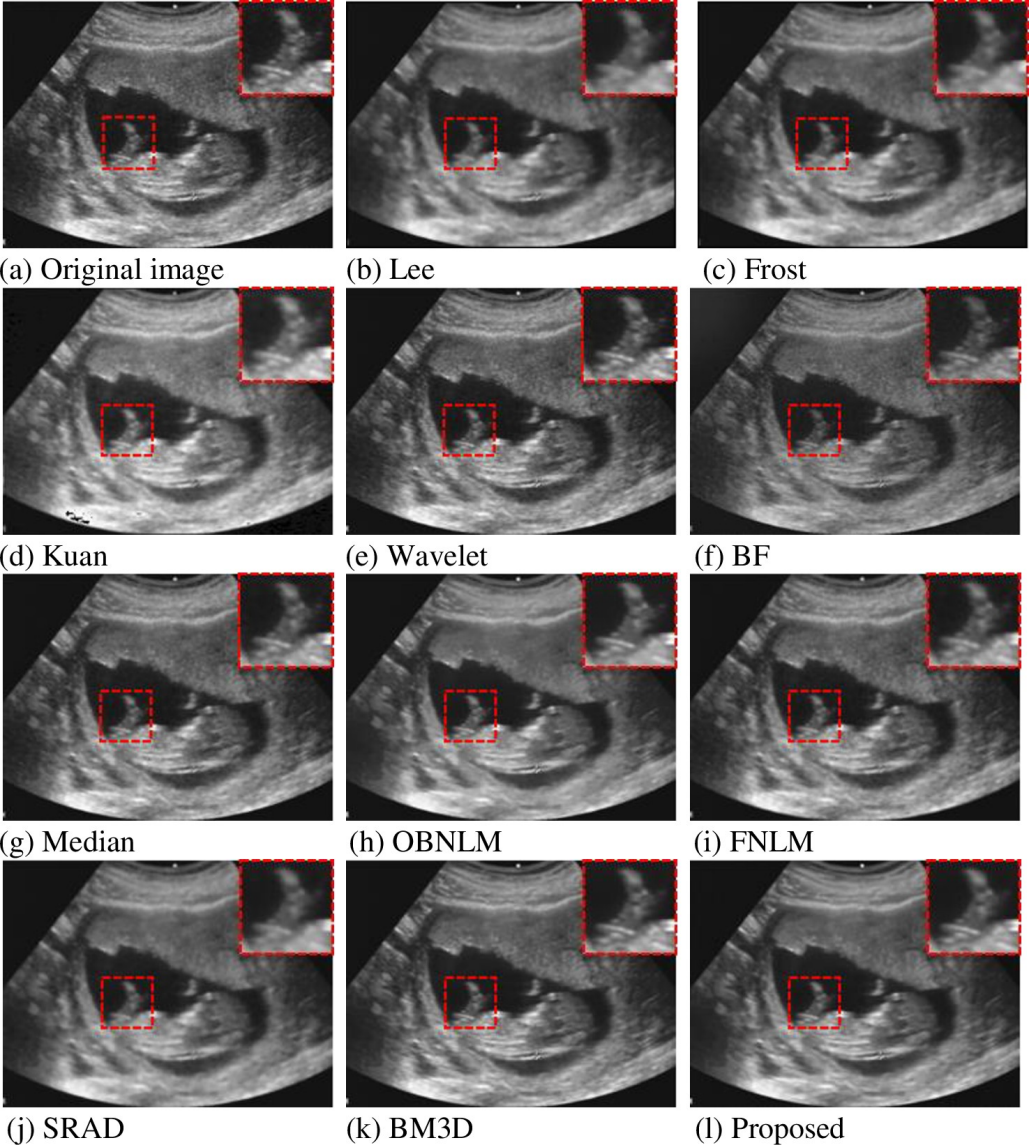
Effect of fetal ultrasound denoising.

**Fig 10:**
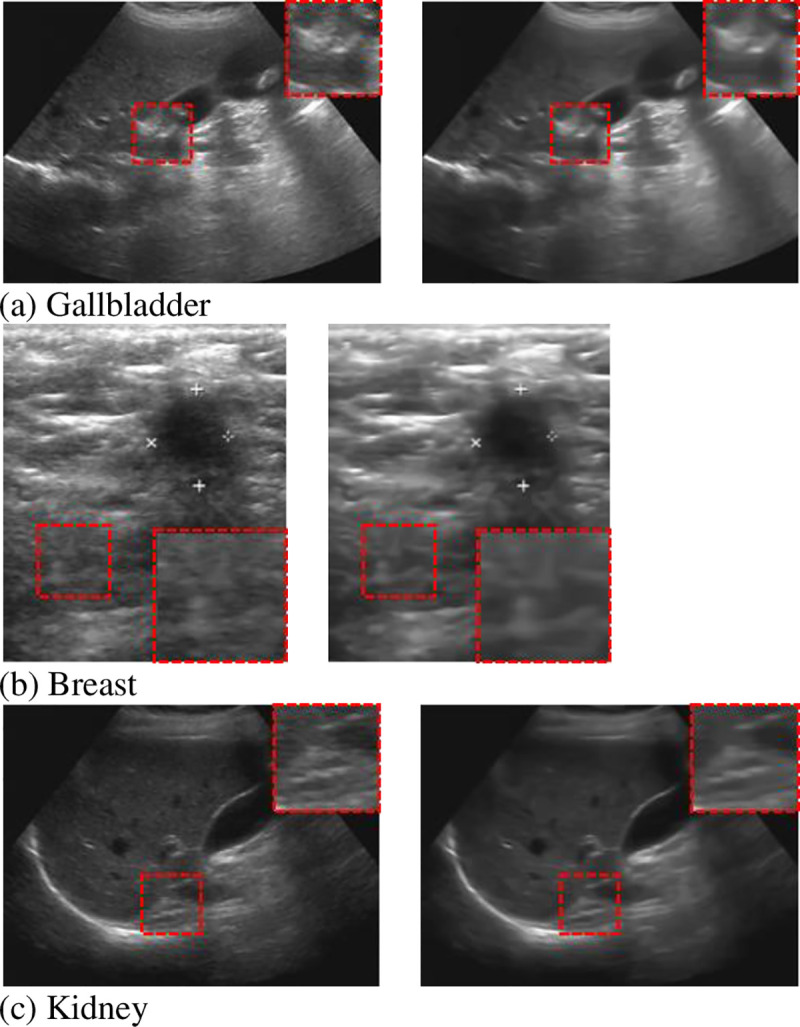
The proposed method to denoise the three organs (left is the original image, and the right is the denoising image).

For the real ultrasound image, it is also based on the adaptive optimization method proposed in Section 3, and the optimal parameter value K = 4 is obtained through the process of Fig 3. From the Fig 10 above, the proposed method has good effects on medical ultrasound images of different organs. Fig 9(A) is a fetal ultrasound image, and Fig 9(E), 9(F), 9(G) and 9(I) have no obvious denoising effect, which is consistent with the simulation results. Fig 9(B), 9(C), 9(D) and 9(H) show denoising while reducing the overall clarity. All other algorithms have good performance: the noise was also considerably reduced, and the edge information could be better preserved compared with the original image. At the same time, for better visual comparison, we framed and selected a specific area for magnified view processing. From a subjective visual point of view, the proposed method also has a good performance.

Given that a noise-free ultrasound image does not exist in reality, the aforementioned reference quality evaluation indicators, such as PSNR and SSIM, are not suitable for evaluation. Therefore, using non-reference evaluation indicators should be considered. Natural Image Quality Evaluator (NIQE) [46], a completely blind algorithm, uses a large number of high-fidelity natural images to extract natural scene statistics in the spatial domain to reflect the quality characteristics of the image. It does not require prior knowledge and image distortion type information, and the evaluation effect is better than full-reference quality evaluation indicators, such as PSNR and SSIM. This evaluation index is in line with the judgment of the human visual system. The higher the NIQE metric, the better the quality of the denoised image. The NIQE value of the denoised image for four sample ultrasound organs images is shown in Table 3.

**Table 3:** NIQE values and average execution time in the four organs images using different methods.

Methods	Fetus	Gallbladder	Breast	Kidney	Average time(s)
Noisy image	4.0832	5.7624	7.0056	6.0639	-
Lee	6.0568	7.5836	7.2054	7.6472	1.097
Frost	5.9347	7.6381	7.4957	7.5951	0.5513
Kuan	4.6768	5.2103	8.057	5.9448	8.4935
Wavelet	4.0774	6.403	7.0107	5.8687	1.8759
BF	4.1489	6.2919	6.6377	6.0692	77.104
Median	5.779	7.3743	8.3701	8.1993	0.4372
OBNLM	5.4198	7.7795	6.5134	9.4745	54.1082
FNLM	5.574	7.641	8.5826	7.8211	2.3097
SRAD	7.347	9.116	8.2652	9.267	2.7201
BM3D	7.5262	8.2179	6.2476	8.8248	6.1307
**Proposed**	**8.2737**	**9.6613**	**8.8068**	**10.6572**	**8.5482**

The result of the above Table 3 shows that the proposed method obtained a satisfactory NIQE value. Thus, compared with other denoising methods, the method in the present work has a better effect in suppressing speckle noise and can produce an image with better quality after denoising. The findings are consistent with the conclusions of the above synthetic graph and simulation graph experiment. It can effectively remove noises of different intensities in real medical ultrasound images, while retaining fine edge structure information, and maintain stability. For the average execution time, the proposed method does not perform very satisfactorily, and it has a slight increase compared to BM3D. Therefore, it is necessary to consider streamlining the calculation in the future to improve the efficiency of the algorithm.

## Conclusions

This work proposes a VIF-based adaptive 2D-VMD ultrasound image frequency division denoising algorithm to address the serious effect of noise on the quality of medical ultrasound images. The main findings are as follows:

1. On the basis of VIF, a method of adaptively determining the parameter K value was designed. This method prevents the under- or over-decomposition of 2D-VMD, which lays the foundation for de-noising after decomposing and strengthens the denoising ability.
2. The information among the sub-images considerably varied after decomposition. The image was divided into low- and high-frequency images, and anisotropic diffusion filter was used to remove a small amount of noise in the low-frequency image, thereby resulting in the maximum retention of low-frequency information. BM3D with strong denoising ability was used to process high-frequency images that protected most edge details while removing a considerable amount of noise.

Various experiments on synthetic, simulated, and real images were performed, and the 10 other denoising methods were compared and analyzed through quantitative evaluation indicators. The experimental results showed that the algorithm has strong denoising ability, is stable in maintaining the edge structure, and has an overall performance that is far better than those of other algorithms. Thus, it can be effectively applied to clinical ultrasound images. The success of the proposed algorithm lays the foundation for the parameter optimization of 2D-VMD and helps to expand the development of denoising ultrasound images in the transform domain.
